# Theory of Mind Deficits and Social Emotional Functioning in Preschoolers with Specific Language Impairment

**DOI:** 10.3389/fpsyg.2016.01734

**Published:** 2016-11-04

**Authors:** Constance Vissers, Sophieke Koolen

**Affiliations:** ^1^Royal Dutch Kentalis, Kentalis AcademySt Michielsgestel, Netherlands; ^2^Behavioural Science Institute, Radboud University NijmegenNijmegen, Netherlands; ^3^Pro Persona for Mental HealthArnhem, Netherlands

**Keywords:** specific language impairment (SLI), social emotional functioning, theory of mind (ToM), neuropsychological functioning, language

## Abstract

Children with Specific Language Impairment (SLI) often experience emotional and social difficulties. In general, problems in social emotional functioning can be cognitively explained in terms of Theory of Mind (ToM). In this mini-review, an overview is provided of studies on social-emotional functioning and ToM in preschoolers (average age from 2.3 to 6.2 years) with SLI. It is concluded that, similar to school-aged children with SLI, preschoolers with SLI have several social-emotional problems and that both cognitive and affective aspects of ToM are impaired in those children. Based hereon, three possible causal models for the interrelation between language, ToM and social emotional functioning are put forward. It is proposed that future research on the construct and measurement of early ToM, social emotional functioning and language development in preschoolers with SLI is needed to achieve early detection, tailored treatment, and ultimately insight into the pathogenesis of SLI.

## Introduction

Children with SLI have often been reported to experience behavioral, emotional and social difficulties (Mawhood et al., 2000; Yew and O'Kearney, 2013; Helland et al., 2014). They have low social self-esteem, poorer social skills and peer relationships and rate themselves as having a higher risk of being bullied (Fujiki et al., 1996; Knox and Conti-Ramsden, 2003; Marton et al., 2005). While behavioral problems appear to decrease during adolescence, emotional problems persist and social problems have even been reported to increase (St Clair et al., 2011). Adolescents and adults with SLI have social emotional problems like low self-esteem and symptoms of anxiety and depression (Howlin et al., 2000; Wadman et al., 2008; Whitehouse et al., 2009; Durkin and Conti-Ramsden, 2010; Conti-Ramsden et al., 2013; Lewis et al., 2016).

Problems in social-emotional functioning can be explained in terms of Theory of Mind (ToM). The concept of ToM was introduced in the 1970s in primate research by Premack and Woodruff (1978), who defined ToM as the ability to represent mental states of oneself and others in order to understand behaviors. Nowadays, distinctive dimensions of human ToM, each with different neuroanatomical underpinnings, can be discerned (Westby and Robinson, 2014). ToM can be explained along cognitive, affective, interpersonal, and intrapersonal dimensions. Cognitive ToM refers to thinking about thoughts, knowledge, beliefs and intentions and affective ToM involves thinking about and experiencing emotions (e.g., Dvash and Shamay-Tsoory, 2014), which can refer either to oneself (intrapersonal) or to others (interpersonal) (e.g., Tine and Lucariello, 2012).

Given the above, it is not surprising that ToM abilities are associated with social emotional maturity and social skills (e.g., Lalonde and Chandler, 1995; Dunn and Cutting, 1999; Carpendale and Lewis, 2004; Caputi et al., 2012). Children with SLI have been reported to have both social-emotional problems and ToM deficits, which bolsters this association (e.g., Andrés-Roqueta et al., 2016). ToM development in SLI is taken to follow a trajectory similar to that in typically developing (TD) children, but at a different pace and with a lower final level of ToM performance (Nilsson and de López, 2016; Spanoudis, 2016). Hence, ToM deficits in SLI continue into adulthood (Clegg et al, 2005; Botting and Conti-Ramsden, 2008).

Studies in typically developing (TD) preschoolers show that important progress in ToM is made during this period. During the second year of life, joint attention, imitation and pretend play develop, which can be taken as evidence for the understanding of others as intentional agents, the ability to form and co-ordinate representations of self and others, and the capacity to form meta representations (Leslie, 1987; Rogers and Pennington, 1991; Tomasello, 1995). At this stage, emotional recognition and mental state vocabulary also start to develop (Astington and Baird, 2005). With a sense of self, children begin to realize that they are separate from others, can have different emotions from others and they start to show empathy by intentionally comforting/helping another person (Thompson and Newton, 2013). Between 4 and 5 years of age, first order ToM, the ability to think about what someone else is thinking or feeling, develops (Wellman et al., 2011).

Up to now, most studies on ToM in SLI have focused on school-aged children. Given the early onset of ToM development, it is surprising that little research has focused on ToM in preschoolers with SLI. Since early childhood is the primary period for both language and ToM to develop, the early development of language and ToM plausibly interact in an facilitative or inhibitory manner. In order to achieve early detection, tailored treatment, and ultimately insight into the pathogenesis of SLI, research on the construct and measurement of early ToM, social emotional functioning, and language development and their existing deficits is necessary. The aim of this review is to provide an overview of state of the art evidence on social functioning and ToM in preschoolers with SLI (average age range: 2.3–6.2 years), to elaborate on theoretical and clinical implications of these empirical data and to give suggestions for future research.

### Social emotional functioning in preschoolers with SLI

Social skills of preschoolers with SLI are shown to be less well developed or at least delayed. For instance, preschoolers with SLI were rated lower by parents and teachers on social competence (e.g., assertiveness, peer social skills) than TD children (McCabe, 2005). Moreover, they were found to be less likely to verbally address other children and to engage more in adjacent rather than sociointeractive play (McCabe and Marshall, 2006). Further, preschoolers with SLI were rated significantly lower by their parents on skills such as cooperation, assertion and responsibility (Stanton-Chapman et al., 2007), although in a later study, language-impaired preschoolers were found to score within the average range (Pentimonte et al., 2016). Andrés-Roqueta et al. (2016) showed young children with SLI to receive a significantly higher number of negative peer-nominations compared to typical children. Likewise, withdrawal was reported as the most frequent problem behavior in language-impaired preschoolers (Maggio et al., 2014).

### ToM in preschoolers with SLI

Deficits in social emotional functioning can be explained in terms of ToM (e.g., Lalonde and Chandler, 1995; Ford and Milosky, 2003; Creusere et al., 2004; Andrés-Roqueta et al., 2016). Below, empirical evidence on ToM deficits in preschoolers with SLI is presented (see Table 1 for an overview of essential aspects of ToM and observed ToM deficits during preschool).

**Table 1:** **Overview of essential aspects of ToM development during preschool age and observed deficits in ToM in preschoolers with SLI**.

**Essential Aspects of ToM development in typically developing preschoolers**		**Observed deficits in ToM in preschoolers with SLI**
Imitation	The expression of the ability to form and coordinate representations of self and others (Rogers and Pennington, 1991). An aspect of interpersonal/intrapersonal cognitive and affective ToM.	Preschoolers with SLI are shown to be less able to imitate prosodic features.
Joint attention	The reflection of an understanding of others as intentional agents (Tomasello, 1995). An aspect of interpersonal/intrapersonal cognitive ToM.	Deficits in joint attention are observed in preschoolers with SLI (and related to that deficits in gestural production and comprehension).
Emotion recognition and understanding	The ability to recognize and understand emotions (Westby and Robinson, 2014). An aspect of interpersonal/intrapersonal affective ToM.	Deficits in understanding emotional meaning are observed in preschoolers with SLI. Findings on emotion recognition are inconclusive, showing typical and impaired performance in preschoolers with SLI.
False belief understanding	The reflection of the ability to see beliefs as mental entities which can deviate from reality and differ between individuals (Wellman et al., 2011). An aspect of interpersonal/intrapersonal cognitive ToM.	Preschoolers with SLI are shown to be impaired in false belief understanding, (related to linguistic functioning of the child).

*See paragraph ToM in preschoolers with SLI for relevant studies supporting these empirical findings*.

### Imitation

Several studies have focused on imitation abilities in preschoolers with SLI. Within a sentence imitation paradigm, Snow (2001) found that although 4-year olds with language impairment imitate rising intonation contours in the same way as TD children, they are impaired in terms of their segmental phonology. Others have shown that children with SLI have more difficulties in imitating sentences with different linguistic and affective intonation contours and with different empathic stress (Van Der Meulen et al., 1997). Hence, language disordered children seemed to be less able to imitate prosodic features, although both children with SLI and typical children were found to show an increase in performance on prosodic imitation and emotion identification with age.

In addition, research has been done on the effectiveness of imitation/modeling procedures for children with SLI. The underlying assumption is that imitation-based interventions should generate language production under control of the clinician aiming to facilitate spontaneous language use (e.g., Camarata et al., 1994). Kouri (2005) studied the effectiveness of modeling (input that requires imitation without any other response requirements) vs. elicitation (input that includes prompts for production) training procedures for late-talking preschoolers with SLI and developmental delay on the production of comprehended lexical items. Overall, it was concluded that both training methods are effective training procedures for preschoolers with language impairment. The exact mechanism through which those procedures facilitate linguistic functioning, however, remains to be specified. Verbal imitation is proposed as the key component, as verbal practice is expected to stimulate language functioning in children who have impaired verbal production. Another explanation would be that the use of minds is what stimulates linguistic functioning in this group of children.

### Joint attention

As far as we know, only a few studies have directly investigated into joint attention in preschoolers with SLI. Farrant et al. (2011) studied the associations between child and maternal socio-emotional engagement, joint attention, imitation and conversation skill in preschoolers with SLI. Deficits were found on all of those skills in these children, compared with TD children. It was proposed that small impairments in parent-child socio-emotional engagement may lead to larger deficits in joint attention, child imitation and conversation skills. In another study (Loveland and Landry, 1986), focus was on attention-directing language and gesture in children with developmental language delay and children with autism. Language delayed children were reported to be better responders to joint attention interactions than autistic children. Both groups of children did not differ from each other on the number of joint attention behaviors, nor on the types of joint attention behaviors used. Gestural behavior of language delayed children was more communicative than that of autistic children. Given the fact that no typical control group was included, no conclusions could be drawn at the level of performance [(mal)functioning] relative to children without autism or developmental language delay.

### Emotion recognition and understanding

A few studies have examined emotion recognition and understanding in preschoolers with SLI. Courtright and Courtright (1983) observed young children with language impairment to perform less well on interpreting vocal cues to affect than typical controls. Similarly, Creusere et al. (2004) examined affect comprehension in young children with SLI using an affect discrimination task and found lower scores for the language impaired group for measures of recognition of facial expressions and nonfiltered speech. The authors argued that children with SLI may miss cues to the emotional state of their conversational partner, which in turn may hamper their understanding of the speaker's communicative intentions. Similarly, other researchers (Ford and Milosky, 2003) found young children with SLI to be able to identify facial expressions, yet, to have problems inferring the appropriate emotion and choosing the corresponding facial expression when presented with an event context. McCabe and Meller (2004) showed no differences between preschoolers with and without SLI on an emotional expression identification test. Language impaired children did, however, perform more poorly on a stereotyped emotional knowledge task, based on which the authors proposed that under certain circumstances children with SLI are impaired in identifying emotions.

### False belief understanding

Various studies have examined false belief (FB) understanding in SLI, some of which have focused on preschool children. Jester and Johnson (2016) showed young children with SLI to perform more poorly on a FB task than their TD peers. Farrant et al. (2006) found impairments in both visual perspective taking and FB understanding in young children with SLI, based on which they propose language to have a facilitating role in ToM. Interestingly, Farrar et al. (2009) found that while syntactic complementation was not correlated with FB performance in preschoolers with SLI, general grammatical development and vocabulary were significant predictors of ToM ability. In line with this, Andrés-Roqueta et al. (2013) found that, compared to age-matched control children, children with SLI showed more problems on several FB tasks; moreover, FB performance in SLI was best predicted by their overall linguistic abilities, and their grammatical abilities in particular. In another study from this group (Andrés-Roqueta et al., 2016), similar results were found; preschoolers with SLI were shown to have a significant delay both in language and performance on FB and strange stories tasks. Several studies (Miller, 2001, 2004; Guiberson and Rodriguez, 2013) found young children with SLI to be able to perform FB tasks with low linguistic complexity, but to show impairments on linguistically more complex FB tasks.

## Discussion

Given the empirical findings presented above, we conclude that preschoolers with SLI have moderate to severe social-emotional problems. ToM deficits can be taken to play an underlying role in these social emotional problems. That is, in preschoolers with SLI impairments in cognitive ToM (imitation, joint attention, false belief understanding) as well as affective ToM (recognizing and understanding emotions) have been found.

The association between social emotional functioning, ToM and language abilities in SLI is not surprising. From early childhood until adolescence, language development and ToM development are entangled (Tager-Flusberg, 2000). Further, the ability to form a ToM is indispensable for mastering language and efficient communication and interaction (e.g., Baldwin and Moses, 2001). Mental representations of one's own and others' inner world are necessary to come to adequate communicative skills. At the same time, language is essential in understanding mental representations and controlling/regulating emotions and thus in mastering ToM (e.g., Dunn and Brophy, 2005; Grazzani and Ornaghi, 2012; Kolk, 2012; Grazzani et al., 2016). Hence, both ToM and language abilities promote social communication, the understanding and regulation of one's own and others' inner worlds and social emotional maturation. Thus, it is not unexpected that children's level of social emotional functioning can be explained in terms of ToM (e.g., Lalonde and Chandler, 1995) and language abilities (e.g., Jenkins and Astington, 1996). Once they emerge, social emotional problems can, in turn, further affect the development of language and ToM. Importantly, deficits in ToM and language cannot account for the full range of social emotional difficulties in SLI. Plausibly, other cognitive functions (such as level of executive functioning) but also environmental factors (such as parental social emotional engagement) influence the development of language and ToM and social emotional maturation (e.g., Leslie, 1987; Bishop, 1997; Cutting and Dunn, 1999; Farrant et al., 2011; Stanzione and Schick, 2014; Vissers et al., 2015).

Social emotional problems in preschoolers with SLI can thus (at least partly) be understood in terms of an interplay between ToM and language, and social emotional problems may in turn further hamper both language and ToM development. The findings presented do not reveal whether ToM impairments cause language impairments or vice versa. Three possible causal models for the relation between language and ToM can be put forward.

According to the first model, ToM facilitates language development. Following this approach, social understanding informs word learning, even in the infancy period (Baldwin and Moses, 2001). ToM is proposed to allow children to learn new words through their sensitivity to referential intentions of others. Accordingly, word learning problems can be explained by ToM deficits (Bloom, 2001; Birch and Bloom, 2002). Bolstering the essential role of ToM in language development, Morales et al. (2000) found that the capacity to respond to joint attention of infants across the first and second year is related to subsequent vocabulary acquisition (see also Mundy and Gomes, 1998).

The second model argues that language fuels ToM development. Stronger relations were found between early language ability and later ToM performance than the reverse, which suggests a causal role for language in ToM development (see Milligan et al., 2007, for a meta-analysis combining results of 104 studies). The importance of language in developing ToM is further emphasized by the finding that deaf children of hearing parents, who typically demonstrate language delays, have ToM deficits, whereas deaf children from deaf families perform identically to same-aged hearing controls on ToM tasks (e.g., Schick et al., 2007). This is explained by assuming that deaf children with deaf parents share a common sign language and are thus exposed to a rich language context. In line with a central role for language in ToM development, Rosenqvist et al. (2014) found language to be the most important predictor (compared to several neurocognitive capacities) of children's emotion recognition ability. There is no consensus on which aspects of language influence ToM development. The semantic approach argues that the development of mental state verbs (e.g., *think* and *feel*) enhances the understanding of own and others' mental representations (Bartsch and Wellman, 1995; Peterson and Siegal, 2000). Others highlight syntactic processing to play an essential role in ToM acquisition (de Villiers, 2007), from the mastering of basic syntax, such as word order (Astington and Jenkins, 1999), to the use of linguistic structures which are embedded or the mastery of syntactic complementation (e.g., De Villiers and Pyers, 2002; Schick et al., 2007). Interestingly, Slade and Ruffman (2005) state that both syntax and semantics contribute to FB understanding. Further, there is substantial evidence for the conversational approach, proposing that ToM development is influenced by conversational interactions about events and aspects of the external world as well as about inner concepts and states. For instance, it has been suggested that parent-child conversations about situations that involve the mind enhance children's understanding of psychological terms and thereby the development of ToM (Turnbull et al., 2009). Talking about the mind is said to promote the differentiation of one's own viewpoint from others' and to stimulate reflection on social and emotional experiences (e.g., Appleton and Reddy, 1996; Symons, 2004; De Rosnay and Hughes, 2006). Bianco et al. (2016) suggest that conversations about the mind promote ToM by enhancing the accuracy of mental-state attributions. Others found that the use and comprehension of meta-cognitive language correlates with FB performance and emotion comprehension (Grazzani and Ornaghi, 2012). Supportive hereof, training 2-year-old children in using mental-state talk appears to enhance ToM (Grazzani et al., 2016). Moreover, engagement in conversations on emotions appears to stimulate ToM (Ornaghi et al., 2014). Emotion understanding can also be enhanced by participation in explanatory conversations (i.e., about emotional reactions) (Tenenbaum et al., 2008). Hence, according to the second model it is language (semantics and syntax but also conversational interactions) that promotes ToM.

According to a third model, language deficits and ToM deficits co-occur because they are driven by a single factor. Both language abilities and ToM abilities could be manifestations of a single neuropsychological underlying structure, for instance working memory (WM) an aspect of executive functioning. Accordingly, various studies have revealed correlations between WM ability and FB performance (e.g., Jenkins and Astington, 1996; Gordon and Olson, 1998), and also between WM and language development (e.g., Adams and Gathercole, 1996; Baddeley et al., 1998; Vissers et al., 2015).

Future research is needed to investigate the nature of the interplay between language, ToM and social-emotional functioning in SLI. Longitudinal designs are helpful to monitor progress in this interplay across the lifespan. As ToM starts to develop already within the first months from birth, at which point linguistic (dis)abilities are still far from clear, longitudinal cohort studies would be of value starting at birth with children at-risk. Further, up to now, most research has focused mainly on aspects of (interpersonal) cognitive ToM. In order to gain more insight into ToM development in SLI, it is necessary to examine interpersonal/intrapersonal cognitive and affective ToM abilities (Westby and Robinson, 2014).

Neuropsychological insight into social-emotional functioning has important clinical implications. The effects of training studies exposing (young) children to ToM vocabulary for instance are promising (e.g., Hale and Tager-Flusberg, 2003; Lohmann and Tomasello, 2003; Bianco et al., 2016). The fact that language and ToM development start in infancy and continue into adulthood implies that to prevent and treat social emotional dysregulations language and ToM interventions should extend into adulthood (see also Stanzione and Schick, 2014).

## Author contributions

Both authors contributed to developing the hypotheses and searched for/studied literature. SK focussed on the empirical part of the mini review. CV integrated all empircal findings, wrote the Introduction and Discussion (conclusions and theoretical/clinical implications) and finalized the review.

### Conflict of interest statement

The authors declare that the research was conducted in the absence of any commercial or financial relationships that could be construed as a potential conflict of interest.
